# Binding Properties of Methyltrimethoxysilane-Modified Silica Sol Particle Surfaces and Their Molecular Dynamics Simulations

**DOI:** 10.3390/ma18132974

**Published:** 2025-06-23

**Authors:** Hongxing Pang, Zhoufu Wang, Hao Liu, Yan Ma, Xitang Wang, Pengcheng Jiang

**Affiliations:** 1 The State Key Laboratory of Advanced Refractories, Wuhan University of Science and Technology, Wuhan 430081, China; phx@wust.edu.cn (H.P.); lh@wust.edu.cn (H.L.); my@wust.edu.cn (Y.M.); wxt@wust.edu.cn (X.W.); 2 School of Materials Science and Engineering, Hunan Institute of Technology, Hengyang 421002, China

**Keywords:** silica sol, modification, binding properties, molecular dynamics, hydrogen bond, network structure

## Abstract

The surface bonding of silica sol particles modified by methyltrimethoxysilane (MTMS) at different temperatures was investigated. Following modification, MTMS hydrolysis products react with silica hydroxyl groups on the surface of silica particles to create a -Si-O-Si-network structure. Additionally, the hydrolysis products formed hydrogen bonds with the silica hydroxyl groups in the silica sol, which strengthened the bonding strength between the silica particles in a synergistic manner. Increasing the modification temperature accelerated the hydrolysis rate of MTMS, promoted the formation of -Si-O-Si-, and enhanced its binding properties. A silica sol model of grafted MTMS was established using molecular dynamics methods at different modification temperatures to explore the effect of hydrogen bonding on the surface bonding of silica sol particles. Ultimately, it was confirmed experimentally that MTMS modification significantly enhanced the bonding strength on the surface of silica particles in silica sols.

## 1. Introduction

Silica sol is a colloidal dispersion of silica nanoparticles in water or organic solvents [1], typically appearing as a light green or milky white transparent liquid. It is chemically inert, odorless, non-toxic, and has good corrosion resistance [1,2]. Silica sol boasts a number of advantageous properties, including a large specific surface area, effective adsorption, high dispersion rate, high temperature resistance, corrosion resistance, and good oxidation resistance [3]. As a result, it is a highly versatile material, finding applications in a diverse range of fields, including textiles [4], coatings [5], casting [6], catalysts [7], and refractories [8].

A substantial number of hydroxyl groups are present on the surface of silica nanoparticles in silica sols. These nanoparticles are highly susceptible to agglomeration and gelation through hydrogen bonding and inter-group interactions [9,10]. The gelation of these substances is influenced by a number of factors, including concentration, electrolyte composition, and surface properties [11,12]. Silica sols can produce bonding with the help of gelation between silica particles [13], but only hydroxyl groups are present on the surface of silica sols and the degree of gelation greatly reduces the effectiveness [8]. Iler et al. [14] observed that when SiO_2_ colloidal particles in silica sols coalesce, siloxane groups (-Si-O-Si-) are formed internally, while externally they exist as silanol groups (-Si-OH). Furthermore, they noted that changes in pH value and the addition of coagulants can affect the coalescence of silica sols. Wang et al. [15] introduced the silane coupling agent 3-methacryloxypropyltrimethoxysilane (KH-570) into the silica sol, which facilitated the formation of an inorganic–organic network structure and enhanced interparticle bonding of the silica particles. It has been demonstrated that grafting organic molecules onto the surface of silica sol particles can significantly promote the formation of a -Si-O-Si- network structure and enhance its usability [15]. Therefore, it is very important to study the surface modification of silica particles in silica sols, especially the surface modification for improving their usability.

The hydrolysis of Si-OR groups on the silane coupling agent reacted with the hydroxyl groups adsorbed on the nanoparticles, resulting in the closure of the active hydroxyl groups and the resolution of the issue of poor dispersion. Concurrently, a Si-O-Si was formed [16,17]. The formation of reticulated polymers with a high degree of cross-linking is facilitated by silane coupling agents with a higher ratio of silicon-oxygen units. Conversely, the hydrolysis of silanes and the water solubility of modification products are adversely affected by the length of the organic chain directly linked to the silicon [18].

Molecular dynamics simulation in research is an effective method to study the properties of composites and the interaction between organic molecules and inorganic nanoparticles. Zhao et al. [19] investigated the effect of different silane coupling agents on the interface between polyimide (PI) and silicon dioxide (SiO_2_) by molecular dynamics. The formation of a thin film between the silane coupling agent, and SiO_2_ enhanced the interfacial bonding energy between PI and SiO_2_ and improved the electrostatic interactions between the PI molecules and surface of SiO_2_. An excess of the silane coupling agent reduced its performance. Wang et al. [20] investigated the thermodynamic properties of SiO_2_-modified cellulose using molecular dynamics, established a different silane coupling agent grafted nano-SiO_2_/cellulose model, and explored the effects of mechanical properties, interaction energy, and hydrogen bonding on the thermodynamic properties. It was found that more hydrogen bonds were formed, and the interfacial bond strength was increased after grafting with coupling agent, which effectively improved the thermal stability of cellulose. Du et al. [21] investigated the sol-gel condensation kinetics of silica glass through molecular dynamics and found that the gelation reaction is driven by the presence of some localized atomic stressors, which are released upon condensation. However, the use of molecular dynamics to study the role of modification temperature in coupling agent-modified silica sols is still limited.

This study utilized methyltrimethoxysilane (MTMS) as a modifier. Initially, the properties of the silica sol before and after modification were assessed to elucidate surface grafting characteristics. Subsequently, the silica sol model of grafted MTMS at varying reaction temperatures was developed through molecular dynamics simulation. The impact of hydrogen bonding on silica sol grafted with MTMS at different temperatures was examined at a microscopic scale. To validate the modification efficacy, silica sol-bonded silicon carbide castables were fabricated and evaluated for their strength upon hardening.

## 2. Materials and Methods

### 2.1. Materials and Reagents

Alkaline silica sol was purchased from Henan Xinmi Mingzhu Waterproof and Anti-corrosion Materials Co., Ltd. (Xinmi, China) with a solid content of 30 wt% and an average particle size of 15.6 nm. MTMS was purchased from Shanghai Macklin Biochemical Technology Co., Ltd. (Shanghai, China) with a purity of 98 wt%. Silicon Carbide (w(SiC) > 96%, 3–5 mm, 1–3 mm, ≤1 mm, 0.074 mm) Purchased from CMT South Handan Wupeng Furnace Lining New Material Co., Ltd. (Handan, China). Silicon powder (0.074 mm) was purchased from CMT South Handan Wupeng Furnace Lining New Material Co., Ltd. (Handan, China). Silicon powder (0.074 mm) was purchased from CMSS Handan Wupeng Furnace Lining New Material Co., Ltd. (Handan, China).

### 2.2. MTMS Modified Silica Sol

At 25 °C, MTMS could not be dissolved in aqueous solution, the system was delaminated and the hydrolysis of MTMS was incomplete. At 50 °C, MTMS was completely dissolved in aqueous solution, and the silanols produced by hydrolysis could be stabilized. Above 60 °C, the proportion of self-condensation between silanols after MTMS hydrolysis was increased with the increase in temperature [22]. Silica sol was modified by adding 0.06 g of MTMS to 10 g of silica sol, stirred for 4 h at varying water bath temperatures (25 °C, 50 °C, 55 °C, 60 °C). The resulting modified silica sol was freeze-dried for 48 h to assess the surface characteristics. The sol refers to silica sol, while sol + M denotes MTMS-modified silica sol.

### 2.3. Characterizations

The viscosities of the silica sols before and after modification were examined using an advanced rotational rheometer (Physical MCR-301, Karlsruhe, Germany) with a shear rate of 0.01~1000 s^−1^ for 180 s and a constant temperature of 25 °C. The morphology of silica sol before and after modification was examined using a high-resolution transmission electron microscope (TEM, 2100 UHR STEM/EDS JEOL, Tokyo, Japan). The chemical bond state on the surface of modified silica sol particles was analyzed using a Fourier transform infrared spectrometer (FTIR, INVENIO R, Karlsruhe, Germany) in the analysis range of 4000~500 cm^−1^. MTMS grafting on the surface of silica sol was determined using a thermogravimetric-scanning differential thermal analyzer (TG-DSC, STA449/6/G, Schlierbach, Germany) in an air environment with a heating rate of 10 K/min and a temperature range of 30~900 °C.

### 2.4. Model Building and Dynamics Simulation Details

The surface of SiO_2_ in silica sol carries a number of hydroxyl groups. During use, hydrogen bonds are formed between SiO_2_ particles through hydroxyl groups, thereby forming a spatial three-dimensional network structure [23,24] and generating bonding strength. Therefore, the number of hydrogen bonds formed is crucial.

**Figure 1:**
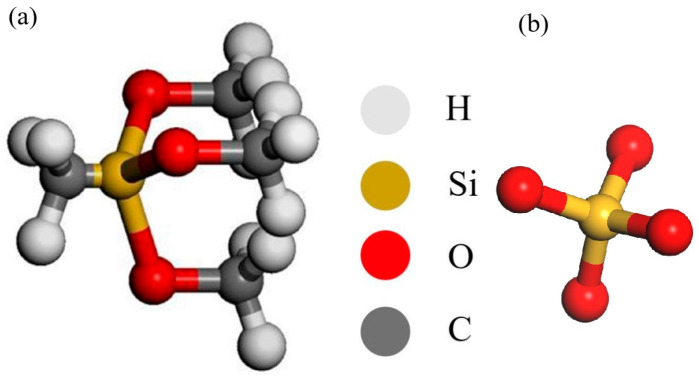
The structure diagram of MTMS and SiO_2_: (**a**) MTMS, (**b**) SiO_2_.

**Figure 2:**
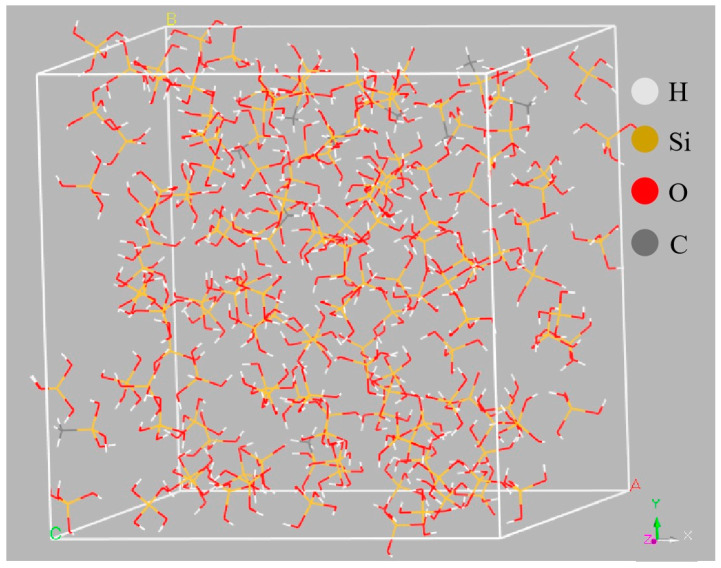
Model of hydroxylated MTMS unreactivity with SiO_2_.

Based on the amorphous cell (AC) module in materials studio (MS), a model of different composites with an initial density of 1 g/cm^3^ was constructed [21], and the model included 130 SiO_2_ and 20 MTMS. The structure diagram of MTMS and SiO_2_ is shown in Figure 1. Since MTMS will be hydrolyzed in the actual reaction process, SiO_2_ needs to be hydroxylated before the model is established [25]. The hydrolysis of MTMS precedes its reaction with SiO_2_ units. Subsequently, the model undergoes geometric optimization over 500 steps using the Forcite module. The unreactioned model is illustrated in Figure 2.

The reaction between MTMS and SiO_2_ was simulated using a Perl script within the Material Studio 2020 software. The simulations were conducted at temperatures of 298 K, 323 K, 328 K, and 333 K, with a distance range of 0.3–0.5 nm and a cross-linking degree of 10%. The COMPASSII force field was employed throughout the simulation, with the Ewald method utilized for electrostatic forces and the Mulliken method for charge distribution. Pressure control was maintained using the Berendsen method with a tolerance of 0.0001 GPa. Additionally, the model underwent cyclic annealing during the reaction to optimize the structural integrity.

### 2.5. Verification Experiment

The silica sol was incorporated into silicon carbide castable by blending the components as outlined in Table 1. Subsequently, the silica sol was added before and after modification, followed by stirring, vibration, and casting to produce a rectangular sample measuring 40 mm × 40 mm × 160 mm. After curing at 30 °C for 24 h and drying at 110 °C for an additional 24 h, the specimens were demolded. The cold compressive strength of the samples was determined using a hydraulic universal press in accordance with GB/T 5072-2008 [26].

## 3. Results and Discussion

**Figure 3:**
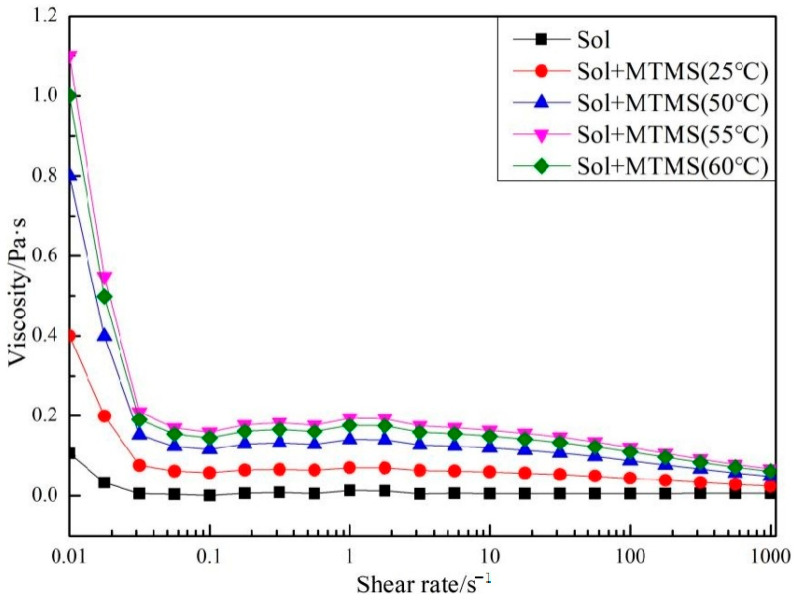
Viscosity with shear rate of colloidal silica and colloidal silica with MTMS.

Figure 3 illustrates the viscosity changes with the shear rate before and after modifying the silica sol. The data indicates that as the shear rate increases, the viscosity of both unmodified and modified silica sol decreases, suggesting a non-Newtonian fluid behavior. This trend is attributed to the behavior of silica particles and the coupling agent under shear stress, leading to dissociation and reconfiguration at the cross-linking points [27]. At higher shear rates, the disconnection rate at these points surpasses the remodeling rate, resulting in decreased viscosity. Additionally, shear may align silica particles and coupling agent molecules along the flow direction, reducing resistance and facilitating relative molecular motion, thereby lowering viscosity [28]. Following modification with the coupling agent, the viscosity of the silica sol increases. The viscosity initially rises and then declines with increasing reaction temperature. This behavior is primarily attributed to the reaction between the hydrolysis products of the coupling agent and the silica hydroxyl groups on the silica particles’ surface in the sol, forming a network structure that enhances viscosity and surface bonding. The variations in viscosity at different reaction temperatures are primarily due to temperature-dependent hydrolysis rates, which subsequently influence the coupling agent’s grafting rate onto the silica particles’ surface in the sol.

Upon introduction into the silica sol, MTMS undergoes hydrolysis as depicted in Equation (1). Under alkaline conditions, this process leads to the substitution of hydroxyl for methoxy groups, reducing the activity of the remaining methoxy groups over a specific duration [29]. Prior to the condensation reaction, OH ions act as catalysts [30], as illustrated in Equation (2). The resulting polymer subsequently interacts with silicon hydroxyl groups on the silica sol particle surface, forming Si-O-Si bonds. This interaction facilitates the development of the Si-O-Si network structure, thereby enhancing the bonding strength among the particles, as demonstrated in Equation (3).

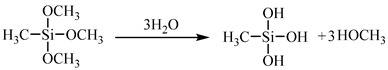
(1)

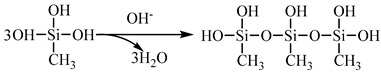
(2)

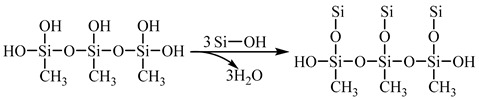
(3)

The optimal reaction temperature significantly influences the hydrolysis of MTMS. Incomplete hydrolysis of MTMS leads to the production of methanol, which can impede the hydrolysis process and result in the formation of a less dense network structure within the silica sol. Consequently, this leads to lower viscosity [23,24,31] and weaker particle-particle binding forces. Conversely, excessively high temperatures can cause rapid volatilization of methanol, accelerating MTMS hydrolysis. This heightened thermal energy promotes intermolecular movement, increasing the likelihood of dehydration between hydroxyl groups. Consequently, this can facilitate condensation reactions between silanols and silica sol [32], diminishing the opportunity for interaction with the silica sol and subsequently reducing viscosity. Ultimately, this decrease in viscosity diminishes the adhesive properties of the silica sol particle surfaces.

**Figure 4:**
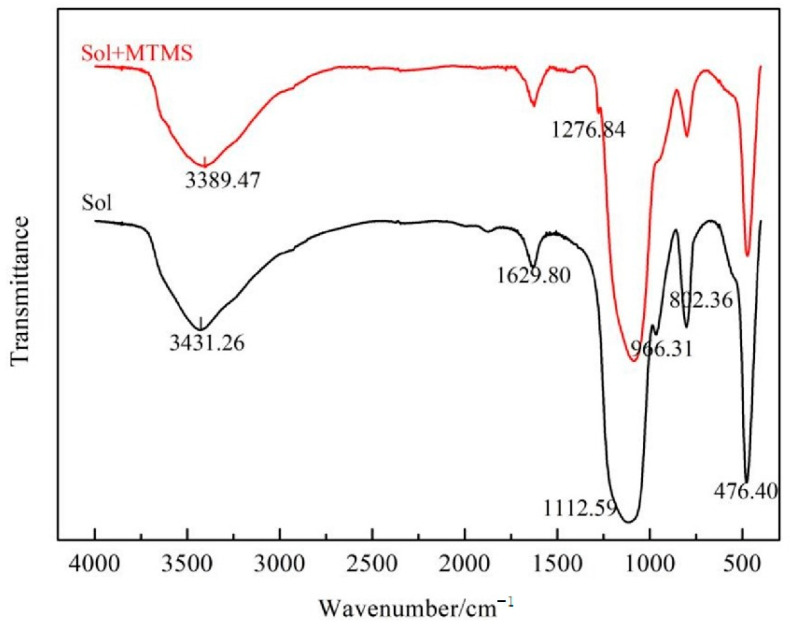
The FTIR spectra of colloidal silica and colloidal silica with MTMS.

In order to verify the modification effect of MTMS on silica sol, the surface of silica sol particles before and after modification was examined. In Figure 4, the infrared spectra of silica sol particles before and after modification are depicted. In the silica sol, characteristic peaks at 3431.26 cm^−1^ and 1629.80 cm^−1^ corresponded to the stretching and the bending vibration peaks of -OH, respectively. Additionally, peaks at 1112.59 cm^−1^ and 802.36 cm^−1^ were identified as the Si-O-Si antisymmetric and symmetric stretching vibration peaks, respectively. The peak at 476.40 cm^−1^ represented the bending vibration peak of the Si-O bond, with a shoulder peak at 963.11 cm^−1^ denoting the Si-OH vibration peak [33,34]. After modification with MTMS, the position of the -OH stretching vibration shifted from 3431.26 cm^−1^ to 3389.47 cm^−1^, and the intensities of the -OH stretching vibration peak at 3431.26 cm^−1^ and the -OH bending vibration peak at 1627.80 cm^−1^ decreased. This suggests an interaction between the hydroxyl functional groups on the surface of silica sol particles and the hydroxyl groups in the MTMS hydrolysate, leading to hydrogen bonding [34]. Comparison of the infrared spectra after the introduction of KH-570 into the silica sol revealed that the Si-OH peak was shifted, but its peak intensity increased, which was analyzed to be caused by the hydrolysis of KH-570 [14]. The characteristic absorption peak of Si-CH_3_ appeared at 1276.84 cm^−1^ [32]. The Si-OH vibrational peak nearly vanished at 963.11 cm^−1^, signifying its reaction with MTMS and the formation of Si-O-Si bonds. Moreover, the peaks at 802.36 cm^−1^ and 476.40 cm^−1^ diminished and broadened, indicating cross-linking of hydroxyl functional groups on the surface of silica sol particles with hydroxyl groups from MTMS hydrolysis, encapsulating the Si-O-Si bonds within the silica sols [32]. This analysis suggests that upon MTMS introduction into the silica sol, hydrolysis-condensation and cross-linking reactions occurred, resulting in the encapsulation of silica sol particles and the creation of an inorganic Si-O-Si skeleton structure containing organic groups.

**Figure 5:**
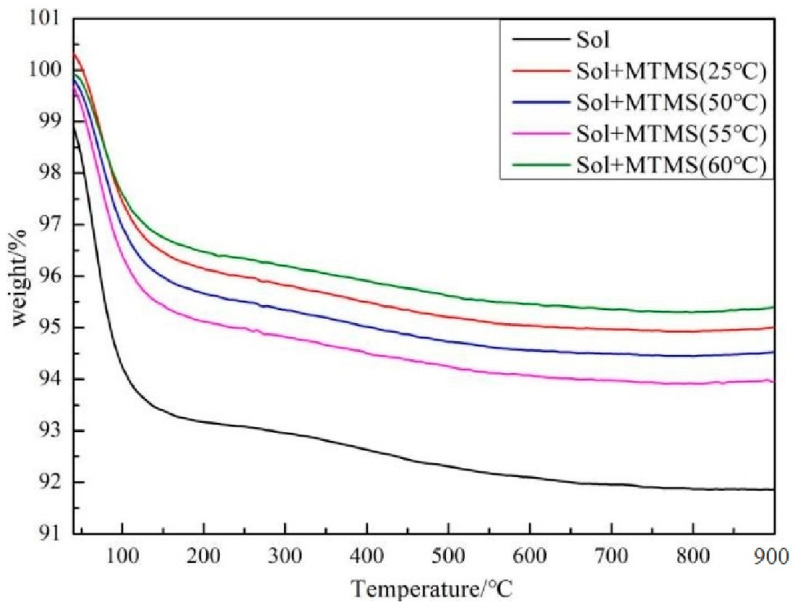
Thermogravimetric curve of colloidal silica and colloidal silica with MTMS.

The thermogravimetric (TG) analysis presented in Figure 5 illustrates the weight loss patterns of silica sol and silica sol treated with MTMS. The weight reduction observed below 200 °C is attributed to the evaporation of physically adsorbed water on the silica sol surface, while weight loss above 200 °C corresponds to the dehydration of hydroxyl, methyl, and other groups [35]. The grafting efficiency of the silane coupling agent was determined from the TG curve. Following the reaction of MTMS with the silica sol, the groups attached to the particles’ surface were identified as -O-Si-(OH)_2_CH_3_ (with a relative molecular mass of 93), whereas the observed thermal weight loss in the TG curve corresponds to -(OH)_2_CH_3_ (with a relative molecular mass of 49). *n_MTMS_*, the grafting rate of MTMS on the surface of silica sol particles, was calculated using Equation (4) [35]:

(4) $$n_{MTMS}=\frac{\frac{\Delta W\times 93}{49}}{W}\times 100\%$$

where ∆*W* is the thermal weight loss, and *W* is the mass before thermal weight loss.

Table 2 displays the grafting rate of MTMS following reactions at various temperatures. The grafting rate initially rises and then declines with increasing temperature. This phenomenon occurs because elevated temperatures can enhance the reaction rate of MTMS with silica sol. However, the heightened temperature also elevates the collision rate among nanoparticles, leading to silica nanoparticle agglomeration. This aggregation impedes further increases in the grafting rate [25]. A higher grafting rate signifies a more complete reaction of MTMS with the silica sol, resulting in the formation of additional Si-O-Si network structures. Consequently, a higher bonding of the silica particles’ surface within the silica sol is achieved.

**Figure 6:**
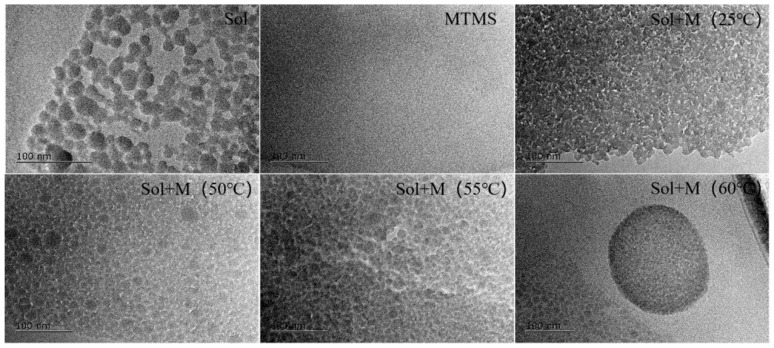
The TEM spectra of colloidal silica and colloidal silica with MTMS.

The silica particles within the silica sol exhibit a uniform distribution. Upon the presence of the hydrolysis product of MTMS, they become interconnected [35,36]. The bonding configuration of the modified silica sol at varying temperatures is depicted in Figure 6. This figure illustrates the absence of silica particle agglomeration, with the MTMS hydrolysis product enveloping each silica particle. The particles are linked through these hydrolysis products, leading to a reduction in intensity and broadening of the Si-O-Si peaks in FTIR analysis upon MTMS introduction. At room temperature, some silica particles may still aggregate, with bonding primarily facilitated by a network structure formed through the condensation of hydroxyl groups on the silica sol particle surfaces. With increasing temperature, MTMS serves as a bridge connecting the silica sol particles. The bonding strength arises from both the condensation network structure of hydroxyl groups and the network structure formed between MTMS and silica sol particles. Excessive temperature, however, results in a non-uniform distribution of MTMS hydrolysis products on silica particle surfaces. This is attributed to incomplete hydrolysis of MTMS at lower temperatures, leading to insufficient hydrolysis product formation that fails to react with the hydroxyl groups on silica sol particles. Temperature does not have much effect on the increase in the hydrolysis rate, but can greatly increase the condensation rate of silanol, the hydrolysis product of MTMS [37]. The MTMS-modified silica sol demonstrates enhanced internal bonding.

**Figure 7:**
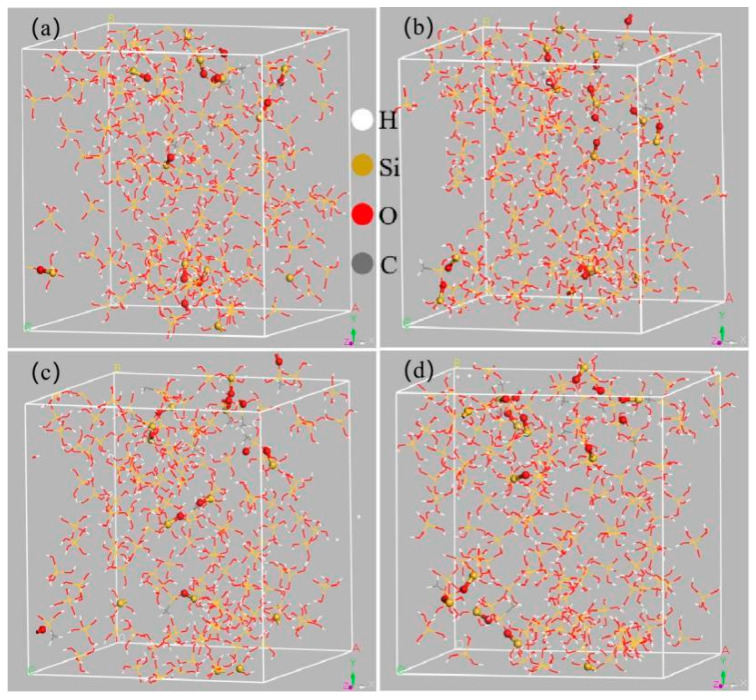
Model of the MTMS reaction with SiO_2_ at different temperature (**a**) 298 K, (**b**) 323 K, (**c**) 328 K, (**d**) 333 K.

Figure 7 illustrates the model post-reaction at varying temperatures. Hydrogen bonding, an intermolecular force, occurs between the silanol groups on the SiO_2_ surface within the silica sol, resulting in the formation of a spatial stereo three-dimensional mesh structure. Consequently, hydrogen bonding plays a crucial role in the formation of the three-dimensional mesh structure in the silica sol [23,24], and the hydrogen bonding network within the polymer significantly influences its binding characteristics [38]. This study primarily focuses on the calculated OH-O hydrogen bonding.

**Figure 8:**
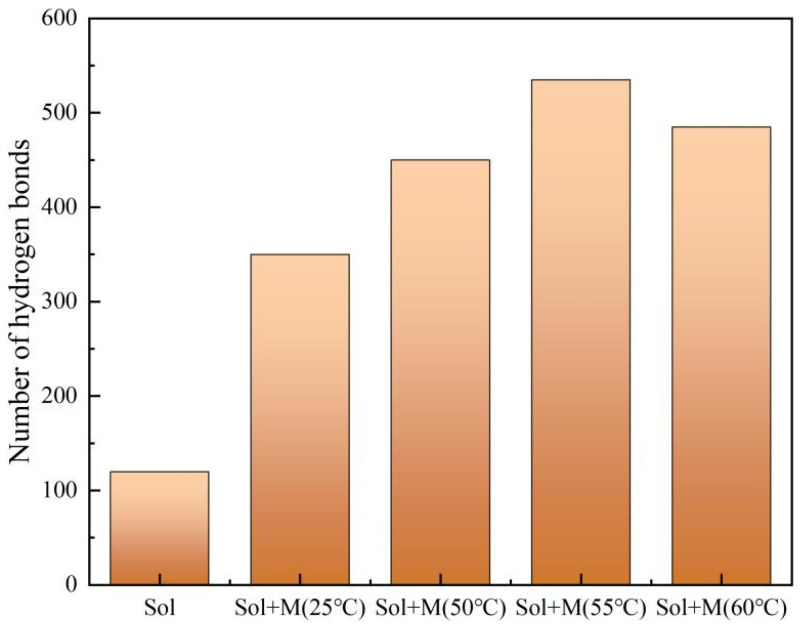
Hydrogen bonds number statistics of different temperatures.

**Figure 9:**
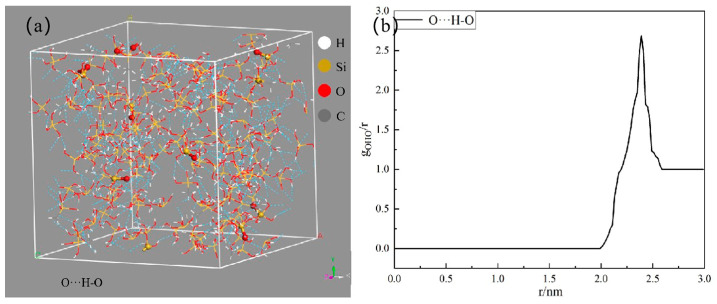
Hydrogen bonds in MTMS grafted silica sol model and radial distribution functions of O···H-O (**a**) Hydrogen bonds, (**b**) radial distribution functions.

Figure 8 illustrates the quantitative analysis of hydrogen bonds within the MTMS and silica structure across varying reaction temperatures. The data reveals a notable increase in the hydrogen bond count following the incorporation of MTMS. Notably, the hydrogen bond count exhibited a trend of initial augmentation followed by a subsequent decline with rising reaction temperatures. The interaction between the silica hydroxyl group in the silica sol and the alcohol group in the MTMS hydrolysis product facilitated the formation of hydrogen bonds post-MTMS introduction, aligning with findings from FTIR analysis. Figure 9 demonstrates hydrogen bonds in MTMS grafted silica sol model and radial distribution functions of O···H-O. The radial distribution function graph shows that the highest peak appears at approximately 0.23 nm, indicating the distance between O···H-O due to hydrogen bonding interactions [39,40,41].

**Figure 10:**
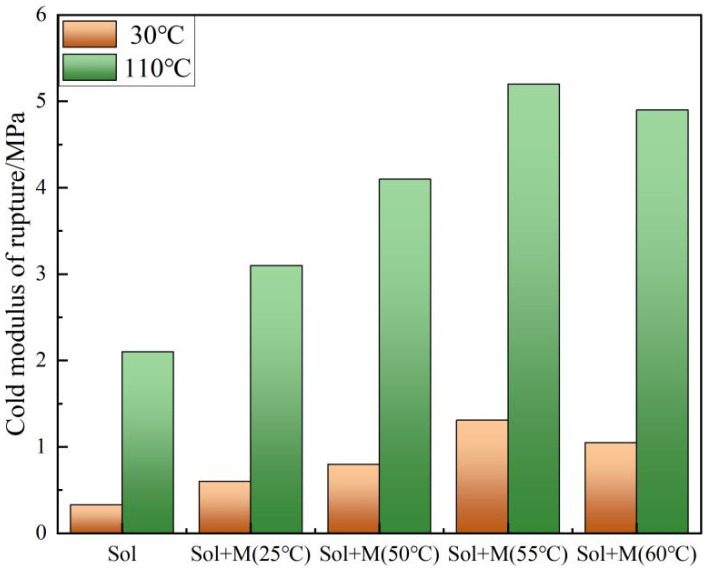
Cold modulus of rupture of a silica sol-bonded silicon carbide castable specimen.

Figure 10 illustrates the demolding strength and dry strength of silica sol-bonded castables. The data indicates that the incorporation of MTMS led to an increase in both demolding and drying strength. These properties are contingent upon the formation of a robust three-dimensional Si-O-Si network within the silica matrix. MTMS facilitates the development of this network, thereby enhancing the material’s overall hardening strength. The relationship between the reaction temperature of MTMS and silica sol and the resulting demolding and drying strength follows a trend of initial increase followed by a decrease, primarily influenced by the grafting rate between MTMS and silica sol. Analysis of the grafting rate data revealed discrepancies, such as a lower grafting rate of 7.84% at 60 °C compared to rates at 25 °C and 50 °C. Despite this, the mechanical strength of the castables was higher, underscoring that the reinforcement of the Si-O-Si network through MTMS is just one factor contributing to the enhanced mechanical properties, with the formation of a hydrogen bonding network also playing a crucial role. In comparison to prior research, the introduction of KH-570 resulted in a 66% strength increase, while MTMS led to a 100% enhancement at room temperature and a remarkable 294% increase at 55 °C. Notably, the addition of MTMS at a lower concentration (0.6%) outperformed KH570 (1%), highlighting the superior strengthening effect of MTMS on silica sol-bonded castables. Therefore, MTMS emerges as a more effective enhancer of strength in these materials.

## 4. Conclusions

This study explores the surface bonding of MTMS-modified silica sols through molecular dynamics simulations, examining the variation in hydrogen bond numbers within MTMS-modified silica nanoparticles at varying temperatures and grafting densities.

The hydroxyl groups present in hydrolyzed MTMS interact with hydroxyl functional groups on SiO_2_ particle surfaces in silica sol, forming hydrogen bonds that enhance inter-particle bonding of SiO_2_. The introduction of MTMS facilitates the reaction of its hydrolysis products with silica hydroxyl groups on SiO_2_ particle surfaces in silica sol, promoting the formation of -Si-O-Si- linkages. Simultaneously, hydroxyl groups in MTMS hydrolysis products establish hydrogen bonds with silica hydroxyl groups, reinforcing the bonding strength through the formation of a hydrogen bonding network. Experimental evidence confirms that the bonding strength between SiO_2_ particles in silica sol is augmented by the synergistic enhancement of the -Si-O-Si- network structure and the hydrogen bonding network structure.

## Figures and Tables

**Table 1:** Main raw material ration of silica sol-bonded SiC castables, w/%.

Silicon Carbide				Silicon Powder	Silica Fume	Silica Sol (20 wt%) (Apposition)
3–5 mm	1–3 mm	≤1 mm	0.074 mm			
21	31	20	13	9	6	9

**Table 2:** The grafting rate of colloidal silica with MTMS.

	Temperature	∆m/%		Grafting Rate/%
		<200 °C	200–900 °C	
Sol	-	6.82	1.31	-
Sol + MTMS	25	3.86	1.13	8.38
	50	4.34	1.14	9.43
	55	4.89	1.16	10.6
	60	3.61	1.11	7.84

## Data Availability

The original contributions presented in the study are included in the article, further inquiries can be directed to the corresponding authors.
